# Supplementary material for: Gnotobiotic rainbow trout (Oncorhynchus mykiss) model reveals endogenous bacteria that protect against Flavobacterium columnare infection
Source: PLoS Pathog. 2021 Jan 29;17(1):e1009302. doi: 10.1371/journal.ppat.1009302 (PMC7875404; doi:10.1371/journal.ppat.1009302)
Supplement: S1 Table — (PDF) [file ppat.1009302.s001.pdf]

Supporting **Table S1. *Flavobacterium* sp. strain 4466 taxonomic identification based on genomic similarities.** The identification was based on whole genome Average Nucleotide Identity (ANI), and percentage of similarity with 16S rRNA and *recA* genes. Whole genome-based bacterial species identification was performed by the TrueBac ID system.

**Taxon**	**ANI (%)**	**ANI coverage (%)**	**16S *rRNA* (%)**	***recA* (%)**
Flavobacterium spartansii	94,65	82,4	97,80	98,51
Flavobacterium tructae	94,62	83,9	97,80	98,11
Flavobacterium chilense	85,26	39,8	97,27	89,48
