# Supplementary material for: Gnotobiotic rainbow trout (Oncorhynchus mykiss) model reveals endogenous bacteria that protect against Flavobacterium columnare infection
Source: PLoS Pathog. 2021 Jan 29;17(1):e1009302. doi: 10.1371/journal.ppat.1009302 (PMC7875404; doi:10.1371/journal.ppat.1009302)
Supplement: S2 Table — (PDF) [file ppat.1009302.s002.pdf]

Supporting Table S2. *Flavobacterium* species genomes retrieved from public databases.

Specie	Assembly	Host	BioSample	FTP
F. tructae	GCF_002217475.1	*Oncorhynchus mykiss*	SAMN06049067	https://ftp.ncbi.nlm.nih.gov/genomes/all/GCF/002/217/475/GCF_002217475.1_ASM221747v1/
F.spartasanii	GCF_002217445.1	*Oncorhynchus tshawytscha*	SAMN06049056	https://ftp.ncbi.nlm.nih.gov/genomes/all/GCF/002/217/445/GCF_002217445.1_ASM221744v1/
F. chilense	GCF_001602525.1	Environment (Loyalsock Creek, USA)	SAMN04506025	https://ftp.ncbi.nlm.nih.gov/genomes/all/GCF/001/602/525/GCF_001602525.1_ASM160252v1/
F. plurextorum	GCF_002217395.1	*Oncorhynchus mykiss*	SAMN06049068	https://ftp.ncbi.nlm.nih.gov/genomes/all/GCF/002/217/395/GCF_002217395.1_ASM221739v1/
F. oncorhynchi	GCF_002217355.1	*Oncorhynchus mykiss*	SAMN06049060	https://ftp.ncbi.nlm.nih.gov/genomes/all/GCF/002/217/355/GCF_002217355.1_ASM221735v1/
F. denitrificans	GCF_000425445.1	*Aporrectodea caliginosa*	SAMN02441540	https://ftp.ncbi.nlm.nih.gov/genomes/all/GCF/000/425/445/GCF_000425445.1_ASM42544v1/
F. cutihirudines	GCF_003385895.1	*Hirudo verbana*	SAMN05444268	https://ftp.ncbi.nlm.nih.gov/genomes/all/GCF/003/385/895/GCF_003385895.1_ASM338589v1/
F. aurantiacus	GCF_000016645.1	NA	SAMN02598357	https://ftp.ncbi.nlm.nih.gov/genomes/all/GCF/000/016/645/GCF_000016645.1_ASM1664v1/
F. hibernum	GCF_000832125.1	Environment (freshwater Antarctic lake)	SAMN02934118	https://ftp.ncbi.nlm.nih.gov/genomes/all/GCF/000/832/125/GCF_000832125.1_ASM83212v1/
F. piscis	GCF_001686925.1	NA	SAMN04570197	https://ftp.ncbi.nlm.nih.gov/genomes/all/GCF/001/686/925/GCF_001686925.1_ASM168692v1/
F. frigidimaris	GCA_900129595.1	Environmental (Antarctic seawater)	SAMN05444481	https://ftp.ncbi.nlm.nih.gov/genomes/all/GCA/900/129/595/GCA_900129595.1_IMG-taxon_2695420960_annotated_assembly/
F. araucananum	GCF_002222055.1	*Salmo salar*	SAMN06049049	https://ftp.ncbi.nlm.nih.gov/genomes/all/GCF/002/222/055/GCF_002222055.1_ASM222205v1/
F. sp. Leaf82	GCF_001422725.1	*Arabidopsis thaliana*	SAMN04151618	https://ftp.ncbi.nlm.nih.gov/genomes/all/GCF/001/422/725/GCF_001422725.1_Leaf82/
F. sp. LM4	GCF_002017935.1	Environmental (Lake Michigan, USA)	SAMN06263772	https://ftp.ncbi.nlm.nih.gov/genomes/all/GCF/002/017/935/GCF_002017935.1_ASM201793v1/
F. pectinovorum	GCF_900142715.1	NA	SAMN05444387	https://ftp.ncbi.nlm.nih.gov/genomes/all/GCF/900/142/715/GCF_900142715.1_IMG-taxon_2698536748_annotated_assembly/
F. sp. GV028	GCF_003386855.1	NA	SAMN08778959	https://ftp.ncbi.nlm.nih.gov/genomes/all/GCF/003/386/855/GCF_003386855.1_ASM338685v1/
