# Supplementary material for: Gnotobiotic rainbow trout (Oncorhynchus mykiss) model reveals endogenous bacteria that protect against Flavobacterium columnare infection
Source: PLoS Pathog. 2021 Jan 29;17(1):e1009302. doi: 10.1371/journal.ppat.1009302 (PMC7875404; doi:10.1371/journal.ppat.1009302)
Supplement: S1 Fig — (PDF) [file ppat.1009302.s003.pdf]

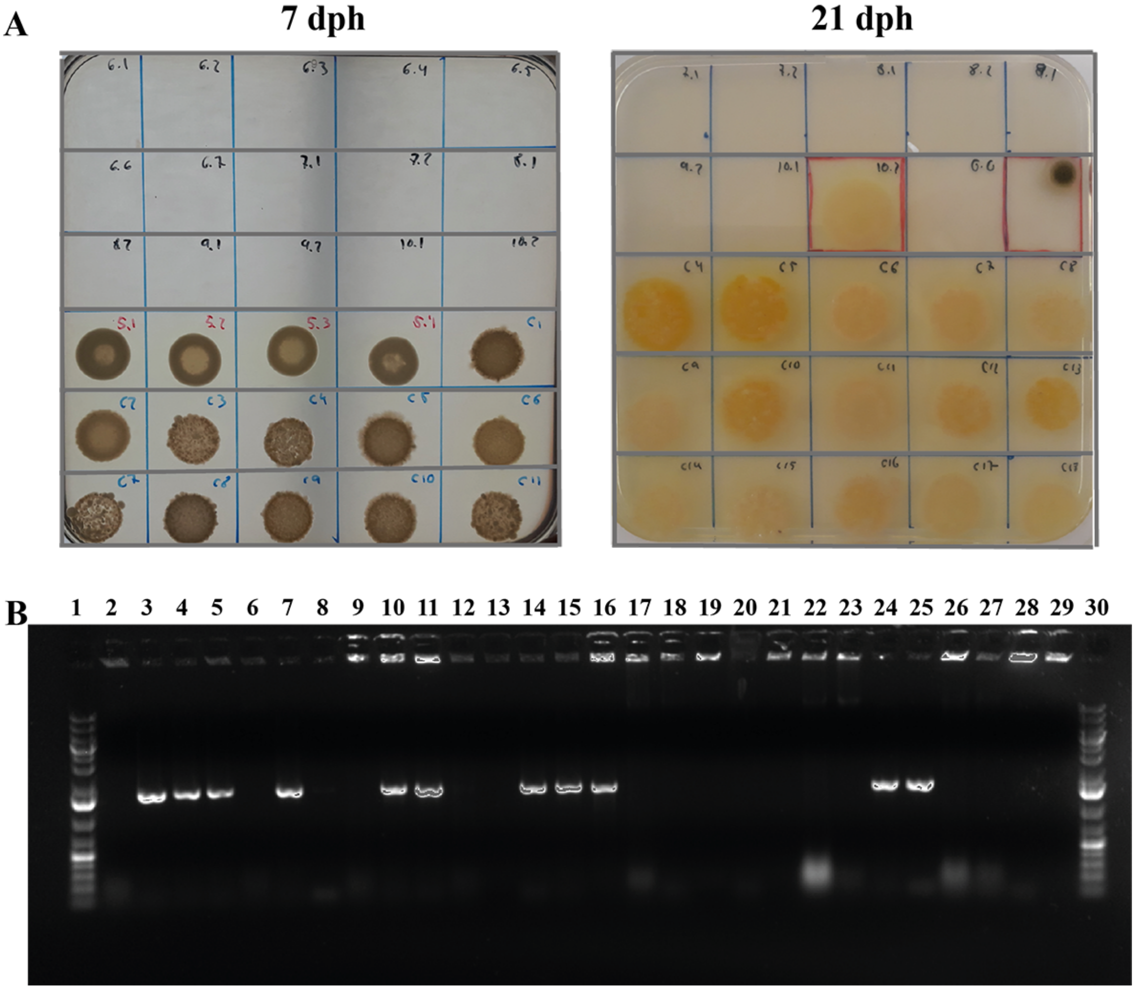

Supporting **Figure S1. Sterility test of rainbow trout larvae raised under GF and Conv conditions**. **A**: Culture-based sterility test of 50 µl samples of rearing water of GF and conventionally reared rainbow trout larvae at 7 and 21 dph. When water samples or fish homogenates showed bacterial CFU on any of the different culture media used, the corresponding animals (or flasks) were considered as non-sterile and removed from the experiment**. B**: PCR sterility test of total DNA extracted from 21 dph GF and conventionally reared rainbow trout larvae and used as a template for amplification of bacterial 16S rRNA gene. This is a representative gel of the PCR results from a simple experiment.Lanes 1 and 30: molecular weight ladder; lane 2: non-template control; lanes 3-5: PCR products from Conv rainbow trout from three different flasks; lanes 6-29: PCR products from GF rainbow trout larvae from 23 different flasks. When water samples or fish homogenates showed a PCR amplification product, the corresponding animals (or flasks) were removed from the experiment.
