# Supplementary material for: Gnotobiotic rainbow trout (Oncorhynchus mykiss) model reveals endogenous bacteria that protect against Flavobacterium columnare infection
Source: PLoS Pathog. 2021 Jan 29;17(1):e1009302. doi: 10.1371/journal.ppat.1009302 (PMC7875404; doi:10.1371/journal.ppat.1009302)
Supplement: S2 Fig — (PDF) [file ppat.1009302.s004.pdf]

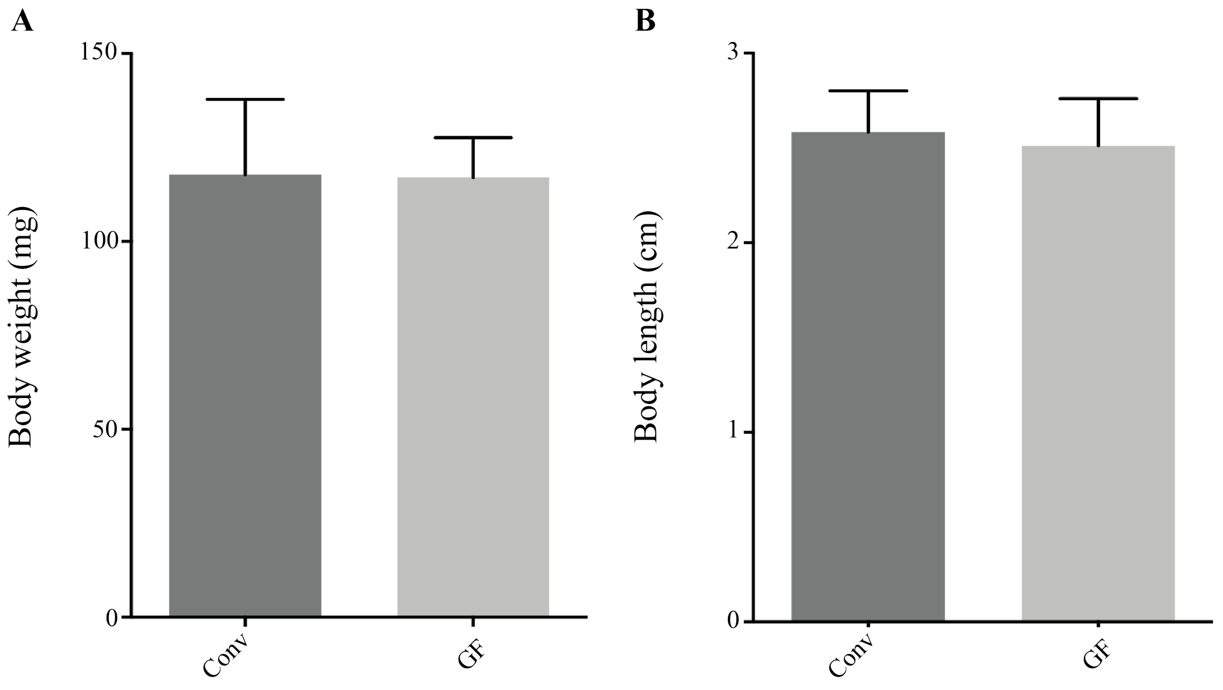

Supporting **Figure S2. Growth performance of rainbow trout larvae raised under GF and Conv conditions.** Conv and GF fish body size (A) and body weight (B) were measured at 35 dph (n= 5).
