# Supplementary material for: Gnotobiotic rainbow trout (Oncorhynchus mykiss) model reveals endogenous bacteria that protect against Flavobacterium columnare infection
Source: PLoS Pathog. 2021 Jan 29;17(1):e1009302. doi: 10.1371/journal.ppat.1009302 (PMC7875404; doi:10.1371/journal.ppat.1009302)
Supplement: S3 Fig — (PDF) [file ppat.1009302.s005.pdf]

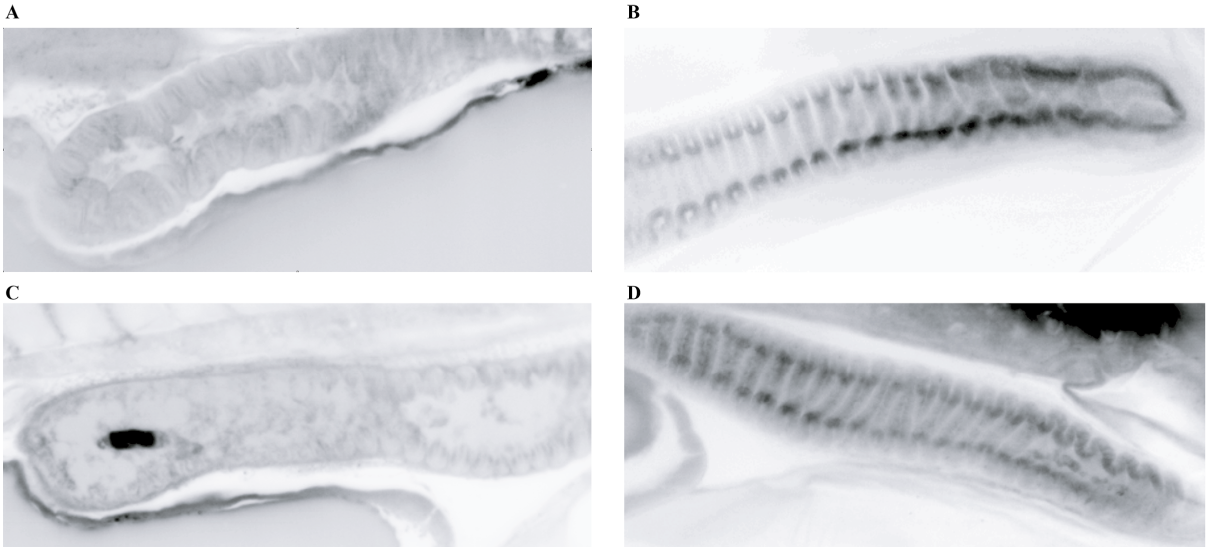

Supporting **Figure S3. Anatomical comparison of the gut of Conv and GF rainbow trout larvae.** 3D deep imaging of whole trout body corresponding to autofluorescence signal acquired by lightsheet microscopy after novel fish clearing processing. Selected optical sections of 21 dph gut were presented for Conv (A and B) and GF (C and D) rainbow trout larvae. Mid-gut (A and C), and posterior gut (B and D). Images representative of two different fish per condition.
