# Supplementary material for: Gnotobiotic rainbow trout (Oncorhynchus mykiss) model reveals endogenous bacteria that protect against Flavobacterium columnare infection
Source: PLoS Pathog. 2021 Jan 29;17(1):e1009302. doi: 10.1371/journal.ppat.1009302 (PMC7875404; doi:10.1371/journal.ppat.1009302)
Supplement: S4 Fig — (PDF) [file ppat.1009302.s006.pdf]

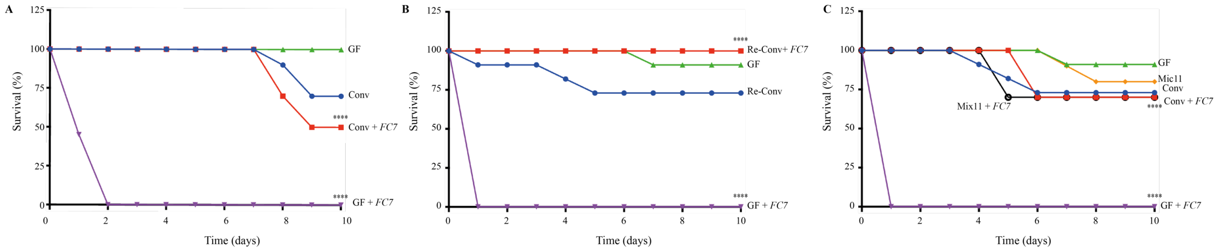

Supporting **Figure S4. Survival of re-conventionalized trout larvae against *F. columnare* Fc7 infection.** Kaplan-Meier graph of GF larvae survival after bath exposure to *F. columnare* strain Fc7, **A:** *F. columnare* strain Fc7 kills GF but not Conv rainbow trout. **B:** GF trout larvae exposed to water used to raise Conv fish at 22 dph show similar survival rates to *F. columnare* infection than Conv trout larvae. **C:** The 11 strains identified from Conv fish microbiota were added to rainbow trout larvae at 22 dph, followed by *F. columnare* infection at 24 dph. This bacterial mixture is able to protect re-conventionalized larvae from infection. Mean and SD plot representing average survival percentage of fish for 10 days after exposition to different pathogenic microorganisms. For each condition n = 10 larvae. All surviving fish were euthanized at day 10 after infection. Asterisks indicate significant difference from non-infected population (****p<0.0001).
