# Supplementary material for: Gnotobiotic rainbow trout (Oncorhynchus mykiss) model reveals endogenous bacteria that protect against Flavobacterium columnare infection
Source: PLoS Pathog. 2021 Jan 29;17(1):e1009302. doi: 10.1371/journal.ppat.1009302 (PMC7875404; doi:10.1371/journal.ppat.1009302)
Supplement: S5 Fig — (PDF) [file ppat.1009302.s007.pdf]

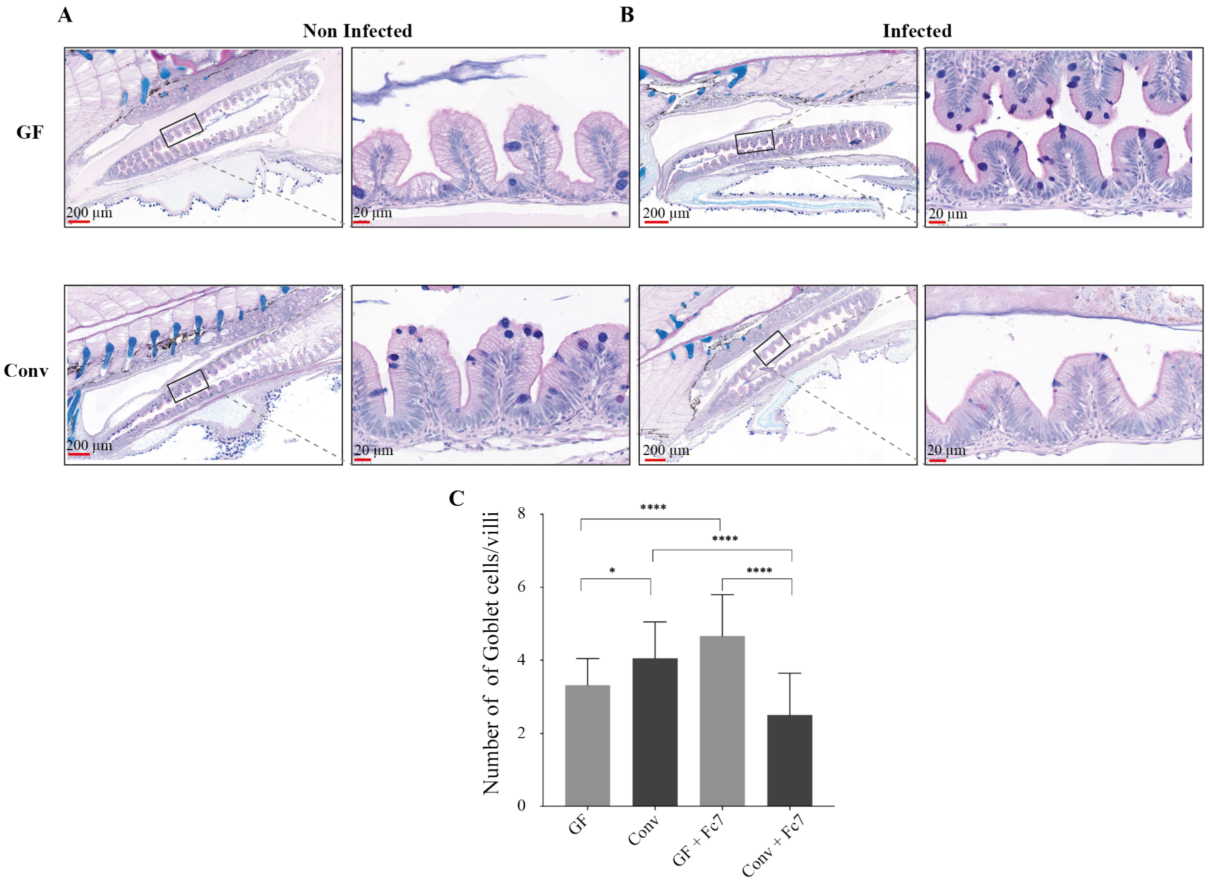


Supporting **Figure S5. Histological comparison of the posterior gut of infected and non-infected Conv and GF rainbow trout larvae. A**: Representative images of intestines of non-infected GF and Conv trout larvae. **B**: Representative images of intestines of infected GF and Conv larvae exposed to *F. columnare* strain Fc7. Fish were fixed for histology analysis at 1 day post-infection (dpi). **C**: Average Goblet cells number per microvilli of posterior gut. Bars represent means ± SD per villi of the same area of posterior gut of 3 fish per condtion. Alcian blue and PAS combined staining of paraffin-embedded rainbow trout larvae for light microscopy. Images and quantification data are representative of three different fish per condition.
