# Supplementary material for: Gnotobiotic rainbow trout (Oncorhynchus mykiss) model reveals endogenous bacteria that protect against Flavobacterium columnare infection
Source: PLoS Pathog. 2021 Jan 29;17(1):e1009302. doi: 10.1371/journal.ppat.1009302 (PMC7875404; doi:10.1371/journal.ppat.1009302)
Supplement: S6 Fig — (PDF) [file ppat.1009302.s008.pdf]

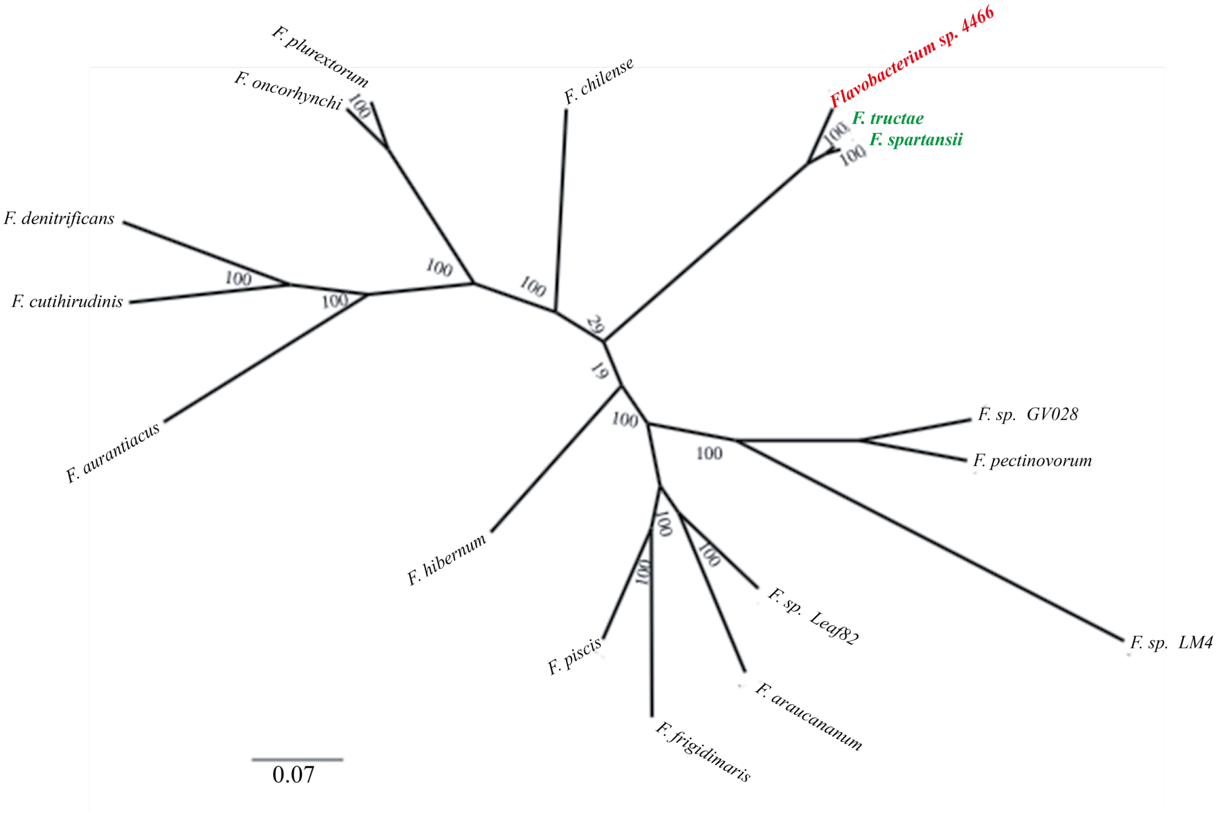

Supporting **Figure S6 Phylogenetic tree illustrating the relationship between *Flavobacterium* sp. strain 4466 and the closest 15 *Flavobacterium* species based on ANI analysis.** The tree was constructed with RAxML (version 8.2.8) by using the 400 most conserved proteins across the proteomes of each strain. Bootstrap support values are indicated in the nodes.
