# Supplementary material for: Gnotobiotic rainbow trout (Oncorhynchus mykiss) model reveals endogenous bacteria that protect against Flavobacterium columnare infection
Source: PLoS Pathog. 2021 Jan 29;17(1):e1009302. doi: 10.1371/journal.ppat.1009302 (PMC7875404; doi:10.1371/journal.ppat.1009302)
Supplement: Attachment — Submitted filename: Perez-Pascual.PPATHOGENS-D-20-01341R.Resp to reviewers.VF.docx [file ppat.1009302.s009.docx]

**Reviewer's Responses to Questions**

**Part I - Summary**
Please use this section to discuss strengths/weaknesses of study, novelty/significance, general execution and scholarship.
 

**Reviewer #1:** *Strengths: directly studies trout, which are an economically important but under utilised experimental system. Logical experiments establish a system using gnotobiotic larvae to model antibiotic-associated opportunistic infections, demonstrate protection by cocktail and then individual species of normal microbiome.

Well written and presented. Missing a few connections (ie CFU recovery) that would strengthen the manuscript by providing additional readouts. Missing mechanistic insight into the mode of protection at this stage but I believe the authors could use the system to rapidly establish a mechanism of protection.*

We thank the reviewer for these comments.

**Reviewer #2:** *This manuscript describes the generation of a gnotobiotic protocol for rainbow trout, and the ability of the trout microbiota to protect against pathogenic bacteria. The paper also describes use of a mock community of trout microbiota that conferred resistance to the infection, and that monoassociations and mock community exposures indicate a specific member of the microbiota produces anti-bacterial activity against the pathogen. This is a significant finding both for trout aquaculture and for gnotobiotic studies in general. As far as I can tell, similar studies have not been done in trout. In general, these experiments were executed well, although I do have some concerns, most of which may be clarified with some editing.*

We thank the reviewer for these comments and we attempted to clarified the manuscript in the submitted revision.

**Part II – Major Issues: Key Experiments Required for Acceptance**
Please use this section to detail the key new experiments or modifications of existing experiments that should be absolutely required to validate study conclusions.

*Generally, there should be no more than 3 such required experiments or major modifications for a "Major Revision" recommendation. If more than 3 experiments are necessary to validate the study conclusions, then you are encouraged to recommend "Reject".*

**Reviewer #1**: *Figure 2 and SFig 4
Need to have at least some basic microscopic analysis of histology. Eg Alcian blue staining for intestinal goblet cell enumeration. Additionally, the SFig4 uninfected samples do not appear well matched.
SFig 4B: there appear to be significant signs of IEC vacuolation and, potentially, mucus staining abnormalities in the CONV-infected (there's something in the lumen). This concern needs to be addressed by higher resolution imaging of more appropriately-stained tissue (Alcian blue, H&E etc)*

We agree and could performed totally new histological analyses on GF and Conv trout larvae infected or not by *F. columnare* FC7. As shown in the new Fig. S5 below, these histology images correspond to longitudinal sections of rainbow trout larvae with much better resolution, which allowed to confirm absence of intestinal damages additional. Following the reviewer recommendation we performed an specific staining (Alcian blue) for Goblet cells quantification and we detected a clear difference in the number of goblet cells in infected GF anf Conv larvae.

Supporting Figure S5. Histological comparison of the posterior gut of infected and non-infected Conv and GF rainbow trout larvae. A: Representative images of intestines of non-infected GF and Conv trout larvae. B: Representative images of intestines of infected GF and Conv larvae exposed to *F. columnare* strain Fc7. Fish were fixed for histology analysis at 1 day post-infection (dpi). C: Average Goblet cells number per microvilli of posterior gut. Bars represent means ± SD per villi of the same area of posterior gut of 3 fish per condtion. Alcian blue and PAS combined staining of paraffin-embedded rainbow trout larvae for light microscopy. Images and quantification data are representative of three different fish per condition.

This is now indicated in the texte

Lines 153-157, Results section:

“Histological analysis performed at 25 dph (24 h post infection) on GF and Conv larvae did not show any sign of intestinal damage (Supporting Fig.S5). However, we observed an increase in goblet cells number in infected vs non-infected GF larvae, whereas Conv infected larvae showed the opposite phenotype when compared to non-infected Conv larvae (Supporting Fig.S5).”

Discussed in the Discussion section

Lines 264-273:

“Although our histology analysis comparing GF and Conv larvae infected or not by *F. columnare* Fc7 did not show any major sign of inflammation of damage, we observed that the number of Goblet cells per crypt increases in infected GF larvae and decresed in Conv larvae. A healthy intestine is determined by biological markers such as Goblet cells count, which secrete mucus with bactericidal properties [42]. Interestingly, a significative decrease in the number of Goblet cells was also observed of non-infected GF larvae compared to those of Conv larvae, as previously reported in zebrafish [37]. The absence of stimulating microorganisms in GF larvae could lead to a dysregulated acute immune response after *F. columnare* infection. These results suggest that the microbiota influence cell differentiation (or maturation) in trout gut epithelium [43], potentially affecting for some aspect of the protection against *F. columnare* infection.”

And accordingly, the Material and Methods section have been updated:

Lines 478-491:

“Sacrificed animals were fixed for 1 week in 4 % methanol-free paraformaldehyde. Whole fixed animals were then dehydrated in a graded series of ethanol solutions (4 hours in 80 % ethanol solution; 4 hours in 95 % ethanol solution, and 4 hours in 100% ethanol). Final dehydration was performed by 100 % xylene solution 2 × 4 hours. Then, samples were embedded in paraffin was solution (3 x 4 hours) and embedded in paraffin wax (3x4h) for polymerization.

Semi-thin sections (thickness 5µm)  were cut with a “Leica Ultracut UCT” ultramicrotome (Leica Microsysteme GmbH, Wien, Austria), and mounted on adhesive slides (Klinipath- KP-PRINTER ADHESIVES). Paraffin-embedded sections were deparaffinized and stained with Alcian Blue (AB) and Periodic-Acid Schiff (PAS) to observe both neutral and acidic mucins and Goblet cells quantification. All slides were scanned with the Pannoramic Scan 150 (3D Histech) and analyzed with the CaseCenter 2.9 viewer (3D Histech). Goblet cells quantification was estimated by manual counting of total AB positive cells in blue per villi of the posterior gut.”

*Figure 4 onwards "mean death day" plots need to be complemented by CFU recovery or bacterial 16s DNA/RNA quantification.*

We understand the reviewer’s concerns, but, the lethality phenotype that we monitored upon *F. columnare* infection results in a rapid tissular degradation which prevent to dissect intestine properly without any external contamination from other organs, in addition to re-performed almost all experiments shown in the manuscript

However, we did integrated this measurement in newly performed experiments included in the revised version of manuscript by adding the in vivo recovery of *Flavobacterium* sp as well as *F. columnare* from the gastrointestinal tract of gnotobiotic rainbow trout larvae, not at time of death, but 24 hours only after infecton from randomly sacrified fish as follows:

Lines 186-192, Results section:

“To evaluate the colonization of gastrointestinal tract by *Flavobacterium* sp. strain 4466 and/or *F. columnare* Fc7, we plated intestines from mono-reconventionalized fish 24 hours after infection in TYES agar after dissection in sterile conditions. Interestingly, whereas both *Flavobacterium* sp. strain 4466 and *F. columnare* Fc7 were able to successfully colonize the gut of mono-exposed rainbow trout (Fig 5C), we only detected to *Flavobacterium* sp. after trout’s infection by *F. columnare* Fc7 (Fig 5C), suggesting a potential competition between both bacterial species.”

Lines 985-987, legend of Figure 5:

C: CFU/mL recovered from dissected intestines from GF fish exposed to F. columnare Fc7, Flavobacterium sp. strain 4466 or both, 24 hours post-infection. (****p<0.0001).

***3. Mechanistic evidence***

*The in vitro data in SFig5 is central to the mechanism of the manuscript and needs to be included as a full figure.*

We agree and followed the reviewer’s proposition by transferring the supplementary Fig. S5 as full new figure 6 in the manuscript.

The in vitro data should be complemented by in vivo CFU recovery showing Flavobacterium colonisation prevents/reduces colonisation of the GI tract by F. columnare.

We agree, please see above: when possible, we complemented the *in vitro* data by adding the *in vivo* recovery of *Flavobacterium* sp as well as *F. columnare* from the gastrointestinal tract of gnotobiotic rainbow trout larvaenot at time of death (recovery impossible) , but 24 only after infecton from randomly sacrified fish as follows:

Fig. 5C

Lines 985-987, legend of Figure 5C:

C: CFU/mL recovered from dissected intestines from GF fish exposed to F. columnare Fc7, Flavobacterium sp. strain 4466 or both, 24 hours post-infection. (****p<0.0001).

Lines 186-192, Results section:

“To evaluate the colonization of gastrointestinal tract by *Flavobacterium* sp. strain 4466 and/or *F. columnare* Fc7, we plated intestines from mono-reconventionalized fish 24 hours after infection in TYES agar after dissection in sterile conditions. Interestingly, whereas both *Flavobacterium* sp. strain 4466 and *F. columnare* Fc7 were able to successfully colonize the gut of mono-exposed rainbow trout (Fig 5C), we only detected to *Flavobacterium* sp. after trout’s infection by *F. columnare* Fc7 (Fig 5C), suggesting a potential competition between both bacterial species.”

*Lines 299-303 suggest mechanism that could be investigated. This paper would be significantly strengthened by the demonstration of at least one of these mechanisms in vivo.*

We cannot agree more, and we did try repeatedly to mutagenize *Flavobacterium* sp. to identify non-protective mutants without success so far. We are currently trying to adapt existing Bacteroidetes/flavobacterial genetic tools to genetically manipulate our specific strain *Flavobacterium* sp.

*Reviewer #2: Supplement figure 5: Was this done in triplicate and these are representative? If so, indicate. If not, these should be repeated.*

Yes, the experiments were done in triplicate and we now clarified this and included this information in the manuscript.

Lines 992-993:

“Representative images of *in vitro* growth-inhibition activity of *Flavobacterium* sp. strain 4466 against different virulent *F. columnare* strains.”

Lines 999-1000:

“This experiment was performed in triplicate.”

**Part III – Minor Issues: Editorial and Data Presentation Modifications**
Please use this section for editorial suggestions as well as relatively minor modifications of existing data that would enhance clarity.

***Reviewer #1:*** *Line 120: Is hatching efficiency directly related to embryo survival or could the increased hatching of GF embryos be due to weakened chorions?*

The hatching efficiency is indeed directly correlated to the number of larvae alive after hatching as not all eggs that hatched led to living larvae. The ratio of survival larvae/eggs number is higher in the case of germ free larvae. While we cannot exclude an impact on chorions weakening after sterilization treatment in the ultimate survival rate, we have no data to support this hypothesis

We now further clarified the notion of hatching survival as follows

Line 119, Results section:

“hatching percentage” instead of “hatching efficiency”.

Lines 373-375, Material and Methods section:

“The hatching percentage was determined by comparing the number of hatched and alive larvae relative to the total number of eggs in the Petri dish.”

*Line 145: 10^7 CFU/ml is a lot of bacteria. Was the mortality rate dose responsive? Surprising that V. anguillarium did not kill larvae.*

The reviewer raises a good point and we indeed did not observe mortality upon *V. anguillarum* infection using our immersion conditions with infection dose used for immersion challenges for other pathogenic bacteria such as *Flavobacterium psychrophilum* (Pérez-Pascual et al., 2017, *Front. Microbiol*. doi: <https://doi.org/10.3389/fmicb.2017.02168>) or *Yersinia ruckeri* (Ohtani et al., 2019. *Aquaculture*. doi: <https://doi.org/10.1016/j.aquaculture.2019.01.041>.

However, it is worth noting that we were looking for radical lethality phenotype and, while it is possible that further optimization of the infection protocol could be required, *V. anguillarum* infection by balneation (immersion with 4x10^6^cfu/ml) only lead to 70 % mortalily at 4 dpi in adult rainbow trout (<https://doi.org/10.1186/s12866-017-1124-1>) and 50% mortality in common soles larvae at 17 dpi with 10^7^ cfu/ml (<https://doi.org/10.1186/s13567-018-0520-3>).

Figure 4 "mean death day" plots: Please show survival curves too for completeness.

We agree and survival plots are now shown as sup. FigS4 (see below)

 Supporting **Figure S4. Survival of re-conventionalized trout larvae against *F. columnare* Fc7 infection.** Kaplan-Meier graph of GF larvae survival after bath exposure to *F. columnare* strain Fc7, **A:** *F. columnare* strain Fc7 kills GF but not Conv rainbow trout. **B:** GF trout larvae exposed to water used to raise Conv fish at 22 dph show similar survival rates to *F. columnare* infection than Conv trout larvae. **C:** The 11 strains identified from Conv fish microbiota were added to rainbow trout larvae at 22 dph, followed by *F. columnare* infection at 24 dph. This bacterial mixture is able to protect re-conventionalized larvae from infection. Mean and SD plot representing average survival percentage of fish for 10 days after exposition to different pathogenic microorganisms. For each condition n = 10 larvae. All surviving fish were euthanized at day 10 after infection. Asterisks indicate significant difference from non-infected population (****p<0.0001).

Line 174: No crucial, but do the Mix11 species colonise at equal proportions? Is there a hint (that could be tied into later observations) that Flavobacteria outperform the other species?

Comparative colonization studies are planed but we did not complete them yet, so we cannot rule out any negative interaction that *Flavobacterium* sp. 4466 over the other members of the Mix11.

Sentence in lines 281-283 does not seem related to the rest of the paragraph.

We agree and we removed this sentence.

Line 292: highlight protection against "multiple" strains of F. columnare in zebrafish embryos.
Related note: the switch to zebrafish embryos for demonstrating protection against multiple pathogen strains was not well justified. Paper would be strengthened by showing protection of trout larvae against multiple pathogenic strains. Is this omission patent related?

We agree and we removed the corresponding zebrafish experiments, to replace them by *in vivo* experiments using infected and mono-reconventionalized GF trout larvae demonstrating that *Flavobacterium* sp. is able to protect rainbow trout against different virulent *F. columnare* strains that are presented in Figure 7 and also in the text as follows:

Lines 215-222, Results section:

“To test whether the protective *Flavobacterium* sp. isolated from the Conv rainbow trout microbiome could protect rainbow trout we re-conventionalized GF fish larvae with *Flavobacterium* sp. 48 hours before exposure to four virulent *F. columnare* strains (Fc7, ALG-00-530, IA-S-4, and Ms-Fc-4) belonging to genomovars I and II, and isolated from different geographical origins and host fish species. *Flavobacterium* sp. strain 4466 conferred protection to rainbow trout larvae against all *F. columnare* strains (Figure 7). Therefore, the *Flavobacterium* sp. strain identified from trout Mix11 is a putative probiotic potentially protecting trout and other fish fom columnaris disease.”

T tests are not appropriate for the multiple comparisons performed in Fig 4 and 5. Reanalyse with ANOVA

We agree with the reviewer since, in Fig 4 and Fig 5, we are comparing different groups (infected and non-infected) of different conditions (i.e. GF, Conv, Reconv-Mix10,…). We now re-analyzed the results of these figures with ANOVA and included the corresponding statistical results in the manuscript. These new statistical analyses have not change the difference previously highlighted in the previous version.

We also updated the Material and Methods section in lines 522-524 as follows:

“Statistical analyses were performed using unpaired, non-parametric Mann-Whitney test or ANOVA using Kruskal-Wallis multiple comparison tests for average survival analysis”

**Reviewer #2:** Minor points:

**Introduction:**
Line 91: “sterile” indicates the fish in those studies had no living organisms. Reference 27 uses antibiotics to kill microbes and only examined growth in aerobic, high nutrient conditions, not by 16S PCR. Check other references for sterility confirmation, or change “sterile conditions” to “reduced microbial load” or “reduced bacterial load”

Since we are citing existing examples of sterily raised fish species, we therefore removed this indeed poorly appropriate reference from the text.

Methods:
Line 335: Were these all from the same clutch of fish, or several different clutches? Given the reference to host genetic background on the ability of the microbiota to protect against pathogens, this could be significant.

The eggs came from different batches obtained from different progenitors with a different genetic background, suggesting that the phenotypes reavealed by our study is somewhat independent of the potential genetic variability between batches.

Line 340: kept at 16C while in petri dishes? Add that detail, or in line 338 that fish were maintained at 16C until the end of the experiment (or whenever their temp was changed).

We added the incubation temperature (16°C) as suggested by the reviewer in line 353.

Also line 340: Is the water tap water, ocean water, what salinity? More details needed.

We used dechlorinated sterile tap water and this is now indicated in lines 353, 354 and 356, in Materials and Methods section.

Lines 341, 348 and elsewhere: how was the water and methylene blue solution sterilized? What type of water was used (instant ocean, tap water, RO water? Is it the autoclaved dechlorinated water from line 340?)

We used autoclaved dechlorinated tap water and the methylene blue solution was filter-sterilyzed on 0.22 µm filters. This information is now included as follows:

Line 362, Material and Methods section:

“in autoclaved dechlorinated tap water, previously filtered with 0.22 µm system.”

Line 343: specify sterile water was added.

This is now specified in line 356.

Line 347: this is at odds with line 335, which says fish were collected at 210-230 degree days. Were only the 210 dd fish derived germ free?

Indeed, thank you for pointing this. We modified the sentence to clarify the eggs age with respect to reception day as follows:

Line 360, Materials and Methods section:

“The eyed rainbow trout eggs received at 210-230 dd were transferred into sterile Petri dishes”

379: add “Following the protocol in [68]. Presence of a band of X bp on an agarose gel indicated contamination and the flask was removed from the experiment.”

We modified the sentence accordingly

Lines 396-398, Materials and Methods section:

“­ following the protocol described in [68], the presence of a band of ~1400 bp on an agarose gel indicated contamination and the flask was removed from the experiment”.

401: Is that 10-12 fish per flask, or is that 5-6 fish in each flask and each flask is in triplicate? This relates to the issues with replication.

As indicated in the text line 420, we used 10-12 fish per flask, considering each flask as a single condition, and we performed experiments at least twice (2 biological replicates using 10-12 fish by flask).

Need to include a method for the microbe-microbe interaction studies.

We thank the reviewer’s comment and we now included the procedure used to study antagonistic microbe-microbe interaction based on an agar overlay growth inhibition assayas follows:

Lines 512-519:

“­ **Agar overlay assay for growth inhibition detection**

The growth inhibitory effect of *Flavobacterium* sp. 4466 has been evaluated using an agar spot test. Briefly, 125 µL from an overnight culture of different strains of *F. columnare* adjusted to OD 1 were mixed to 5 mL of top agar (0.7 % agar) and overlaid on plates of TYES agar. Five µL of overnight culture of *Flavobacterium* sp. 4466 were then spotted on the overlay of targeted bacteria. The plates were incubated at 28°C for 24 h. Growth inhibition of *F. columnare* was recorded by observation of a clear halo surrounding *Flavobacterium* sp. colony. Sterile TYES broth was used as a mock and the experiment were performed in triplicate”.

**Results:**
In some places the manuscript states fish were reconventionalized at 21 dph, others it’s 22 dph. Be consistent.

We agree, this was a mistake and germ-free fish were re-conventionalized using conventional fish-tank water or well identified bacterial suspensions at 22 dph. This is now corrected throughout the text.

Figure 1: Label the timeline with “dph”

This now labelled in Figure 1

Table 1: add the identification of the strains so that if people contact you to get the strains, they can give you the correct strain identification. For example, if I contacted you and asked for Aeromonas rivipollensis 1, how would you ensure that I received the strain from this paper? Related, indicate whether you deposited the strains to a repository.

We thank the reviewer for drawing our attention on this practical issue. We now included an additional indication corresponding to our laboratory collection identification code.

Supplement figure 1 and elsewhere: Is figure S1 a representation of the PCR gels used, or is that all the flasks that were used in these experiments?

The agarose gel showed in Figure S1 is representative of the PCR gels used as an example of the procedure followed for sterility screening for each experiment. As suggested by the reviewer, we specify that

“this is a representative gel of the PCR results from a simple experiment” (line 1051).

It appears that there were 16 germ free flasks; if the pathogen exposures were done in triplicate flasks of 10 fish each (not sure how you would get the error bars in figures 3 & 4 without that) that would be 15 of the 16 flasks, not enough flasks left for the Re-conv, mix 11, etc experiments. State that the PCR gel is representative (if it is) of the PCR results and clarify how many flasks were used for each experiment.

As indicated in the text, in lines 373 and 420, for each experiment we used 10-12 larvae per flask, considering that each flask corresponds to an specific conditions ( i.e. Re-conv, Mix11, among others).

Supplement figure 4: Can’t read the scale bars to determine whether the images are at the same scale.

We agree and produce a totally new set of histology analysis analyses on GF and Conv larvae infected or not by *F. columnare* Fc7 on which scales bars are more clearly indicated

Supporting Figure S5. Histological comparison of the posterior gut of infected and non-infected Conv and GF rainbow trout larvae. A: Representative images of intestines of non-infected GF and Conv trout larvae. B: Representative images of intestines of infected GF and Conv larvae exposed to *F. columnare* strain Fc7. Fish were fixed for histology analysis at 1 day post-infection (dpi). C: Average Goblet cells number per microvilli of posterior gut. Bars represent means ± SD per villi of the same area of posterior gut of 3 fish per condtion. Alcian blue and PAS combined staining of paraffin-embedded rainbow trout larvae for light microscopy. Images and quantification data are representative of three different fish per condition.

Supplement figure 5: Scale bars?

Following the reviewer’s comment, we introduce the scale bars for each panel (now figure 6).

Line 188: “Consistently” – how many times was the sequencing performed and/or reanalyzed? This just seems like an odd word here.

This was indeed unclear and we replaced “*Consistently*” by associated

Lines 196-198:

“We identified a cluster of 12 genes potentially associated to this phenotype in the *Flavobacterium* sp. strain 4466 genome (*tssB, tssC, tssD, tssE, tssF, tssG, tssH, tssI, tssK, tssN, tssP* and *tssQ*) characteristic of type 6 secretion system (T6SS)”.

**Discussion:**
Line 242: Your fish were not GF up to 35 dph. They were GF from -5dph to 35 dph. As far as I can tell, your fish were conventional for 210-230 degree days before they were treated. Fish undergo major development while in their corions.

The reviewer is right and we modified the sentence as follows:

Line 246: “our approach produced larvae that were GF from hatching to 35 dph*”.*

Major concerns:

It is not clear what the biological replication of the experiments are. For example, in the pathogen exposures: how many flasks of fish were exposed to the pathogens? For examples of this, see line 916, 144-148, 159-161, 171-175. Line 402 indicates that each experiment was repeated at least twice; does that mean from derivation of germ free fish to termination of the experiment, or does that mean the germ free fish were divided and at least two flasks were used in each experiment?

As indicated in the text, we used 10-12 fish per flask, considering each flask as a single condition, and we repeated the experiments at least twice (2 biological replicates using 10-12 fish by flask). Regarding the specific example described by the reviewer, we used one flask containing 10-12 fish for each exposure to pathogenic strain, and this experiment was independently repeated twice (two biological repetitions).

Concerning the sentence in line 402 (now line 421), it means that the experiments were repeated independently from derivation of germ-free fish to the end of the infection challenges.

Lines 120-122 and elsewhere: Were conventional eggs made germ free and then microbes were added, or were they untreated (that is, didn’t go through the germ free derivation protocol)? If the latter, the process of deriving eggs germ free may have affected the fish in lines 120-122 and elsewhere.

As indicated line 121, the conventional eggs were not treated at all and they were kept in initially sterile dechlorinated water from their acclimation to their hatching, in the same way that treated GF eggs were handled. In order to be clearer, we added the following sentence in Material and Methods section:

Lines 375-376: “Conventionally raised eggs followed the same procedure from their acclimation until hatching without expose them to the sterilization protocol mentioned above*”.*

Concerning the possibility of GF fish higher sensibility due to the sterilization treatment, it is an option that we cannot exclude. However, GF fish are not sensitive to all tested fish pathogens and when GF fish are re-conventionalized with conventional fish rearing water before infection, we restored the fish resistance to *F. columnare* infection.”

These results therefore rather suggest the GF fish sensibility to the infection is associated to the absence of a protective microbiota:

Line 121-122: “Possibly due to higher susceptibility of Conv eggs to….” This is speculative. A number of things can effect hatching efficiency.

We agree with the reviewer that a number of factors can affect conventional hatching efficiency, and we toned down this sentence by presenting other potential explanations for increased susceptibility of convention,al eggs

Lines 120-123 :

“The lower hatching efficiency of Conv eggs could be due to a different factors such as physico-chemical parameters modification induced by endogenous microbiota (i.e. dissolved O_2_ concentration or water pH), or higher susceptibility to opportunistic infections.”

Regarding the iDISCO, 3D imaging, and histology (described in results in 131-138 and histological results in lines 153-154): What measurements were taken? What were the results from the measurements?

Both iDISCO and histological analyzes were meant to be qualitative and not quantitative.

Was the person doing the analysis blind to the treatment? “

No, unfortunately, the persons in charge did not perform a blind analysis

No damage” doesn’t speak to what aspects of inflammation/lesions/delayed growth/color, etc, was measured and used to determine there was no damage or delay in development.

We agree and performed totally new histological analyses on GF and Conv larvae infected or not by *F. columnare* FC7. As you could see in the new Fig. S5 below, these histology images have much better resolution which allowed to confirm absence of intestinal damages additional. However, we noticed a difference in the number of goblet cells in infected GF larvae (compare to GF), by contrast with a reduction of the number of goblet cells in infected Conv larvae.

This now indicated in the text

Lines 154-157, Results section:

“Histological analysis performed at 25 dph (24 h post infection) on GF and Conv larvae did not show any sign of intestinal damage (Supporting Fig.S5). However, we observed an increase in goblet cells number in infected *vs* non-infected GF larvae, whereas Conv infected larvae showed the opposite phenotype when compared to non-infected Conv larvae (Supporting Fig.S5)”.

Discussed in the Discussion section

Lines 264-273:

“Although our histology analysis comparing GF and Conv larvae infected or not by *F. columnare* Fc7 did not show any major sign of inflammation of damage, we observed that the number of Goblet cells per crypt increases in infected GF larvae and decresed in Conv larvae. A healthy intestine is determined by biological markers such as Goblet cells count, which secrete mucus with bactericidal properties [Elsabagh, 2018]. Interestingly, a significative decrease in the number of Goblet cells was also observed of non-infected GF larvae compared to those of Conv larvae, as previously reported in zebrafish [Rawls, 2004]. The absence of stimulating microorganisms in GF larvae could lead to a dysregulated acute immune response after *F. columnare* infection. These results suggest that the microbiota influence cell differentiation (or maturation) in trout gut epithelium [Bates, 2006], potentially affecting for some aspect of the protection against *F. columnare* infection.

.”

And the Material and Methods section have been updated accordingly:

Lines 478-491:

“Sacrificed animals were fixed for 1 week in 4 % methanol-free paraformaldehyde. Whole fixed animals were then dehydrated in a graded series of ethanol solutions (4 hours in 80 % ethanol solution; 4 hours in 95 % ethanol solution, and 4 hours in 100% ethanol). Final dehydration was performed by 100 % xylene solution 2 × 4 hours. Then, samples were embedded in paraffin was solution (3 x 4 hours) and embedded in paraffin wax (3x4h) for polymerization.

Semi-thin sections (thickness 5µm)  were cut with a “Leica Ultracut UCT” ultramicrotome (Leica Microsysteme GmbH, Wien, Austria), and mounted on adhesive slides (Klinipath- KP-PRINTER ADHESIVES). Paraffin-embedded sections were deparaffinized and stained with Alcian Blue (AB) and Periodic-Acid Schiff (PAS) to observe both neutral and acidic mucins and Goblet cells quantification. All slides were scanned with the Pannoramic Scan 150 (3D Histech) and analyzed with the CaseCenter 2.9 viewer (3D Histech). Goblet cells quantification was estimated by manual counting of total AB positive cells in blue per villi of the posterior gut.”

Lines 339-340: Were the petri dishes with treated fish sealed to ensure that microbes weren’t able to get into the petri dishes? Fungi in particular are able to get into unsealed petri dishes. These would not be detected by plating at 16C for 2 days or by 16S sequencing.

Considering the long incubation period from eggs acclimation to the last egg hatching (around 6-7 days) and the eggs and larvae oxygen needs, we left the plate unsealed in order to allow a continuous air exchange.

Nevertheless, we also evaluated eventual fungi or yeast contaminations by plating at 16°C for 2 to 5 days allowing us to detect bacterial as well as fungal contaminations (please, see the Supplementary Figure 1A).

The methods for the germ free derivation and the fish handling are confusing. I would recommend combining the two into one section called “Sterilization and husbandry of fish”.

We understand but the first one correspond to eggs handling and the second section to the handling of fish once hatched. We therefore consider that this correspond to two different Material and Methods sections.

Line 916 and 144-148: Was the water tested after the exposure and at the end of the experiment to ensure these were monocultures?

Yes, we always tested the water at the end of each experiment to ensure either that the fish were sterile (if germ-free) or that only one bacterial colony morphotype (fish mono-reconventionalyzed) was detected after plating on agar plates.

Line 401 indicates specific media was used for each pathogen; if these are selective media, other microbes that contaminate the water/fish would not be detected. Yersinia ruckeri, V. anguillarum, and L. garvieae were all grown at 28C. F. columnae, Chryseobacterium massiliae and F. psychromphilum were grown at 18C. It could be assumed that most of the general microbiota and other microbes that may contaminate the flasks grow at 16C. These temperature differences may inhibit the ability to detect contaminants in the cultures with the pathogens or other microbe exposures.

We used a combination of non-selective culture media (TYES, LB, TSB or BHI) to grow the bacteria investigated in this study. This allowed us to probably detect all the bacteria used in our study, although not always on the same plates. Regarding the detection of external contaminants, these media are routinely used to allow the growth of a large spectrum of microorganisms.

Concerning the growth temperature, we indicated that we monocultured each pathogen at their optimal growth temperature. However, for axenic or re-conventionalized fish infections, all animals were kept at 16°C, the growth temperature adapted to the rainbow trout. Consistently, as fish general microbiota and other microbes that may contaminate the flask grow at 16°C, the sterility tests were always performed at 16°C.

Reviewer #1: **Yes:** Stefan Oehlers

Reviewer #2: No
